# Supplementary material for: Association Between Differential Heterogeneity of Antibiotics Consumption and Share of Resistant Pathogens and Its Implication for Antibiotic Stewardship in a German Hospital Intensive Care Unit
Source: Antibiotics (Basel). 2025 Dec 15;14(12):1266. doi: 10.3390/antibiotics14121266 (PMC12729597; doi:10.3390/antibiotics14121266)
Supplement: Supplementary file 1 [file antibiotics-14-01266-s001.zip › antibiotics-3980679-supplementary.pdf]

Supplementary Materials to

Association Between Heterogeneity of Antibiotics Consumption and Share of Resistant Pathogens and its Implication for Antibiotic Stewardship in a German Hospital Intensive Care Unit

by Hans H. Diebner[1*], Pierre Schumacher[1] , Tim Rahmel[2] , Michael Adamzik[2], Nina Timmesfeld[1†], Hartmuth Nowak[3†]

[1*] Ruhr-University Bochum, Department for Medical Informatics, Biometry and Epidemiology, Bochum, 44801, Germany.

[2] Ruhr-University Bochum, Knappschaft Kliniken University Hospital Bochum, Department of Anesthesiology, Intensive Care Medicine and Pain Therapy, Bochum, 44892, Germany.

[3] Ruhr-University Bochum, Knappschaft Kliniken University Hospital Bochum, Department of Anesthesiology, Intensive Care Medicine and Pain Therapy, Center for Artificial Intelligence, Medical Informatics and Data Science, Bochum, 44892, Germany.

[*] Corresponding author(s). E-mail(s): hans.diebner@ruhr-uni-bochum.de

[†]These authors contributed equally to this work.

Supplement to Section 2.1.2 of the main document:
Documented Antibiotic Administration

In addition to the total consumption of the 7 most frequently used antibiotics over the entire observation period as shown in section 2.1.2 of the main document, Figure S1 shows the monthly aggregated consumption of these antibiotics. Of particular interest is the consumption gradually declining towards the end of the observation period of ciprofloxacin, which can be explained by the safety concerns raised in a red-hand letter from the competent authorities. Moreover, during the second half of the observation period, meropenem (a last resort carbapeneme antibiotic) apparently rapidly catches up with piperacillin (a broad-spectrum ß-lactam antibiotic) in terms of importance.

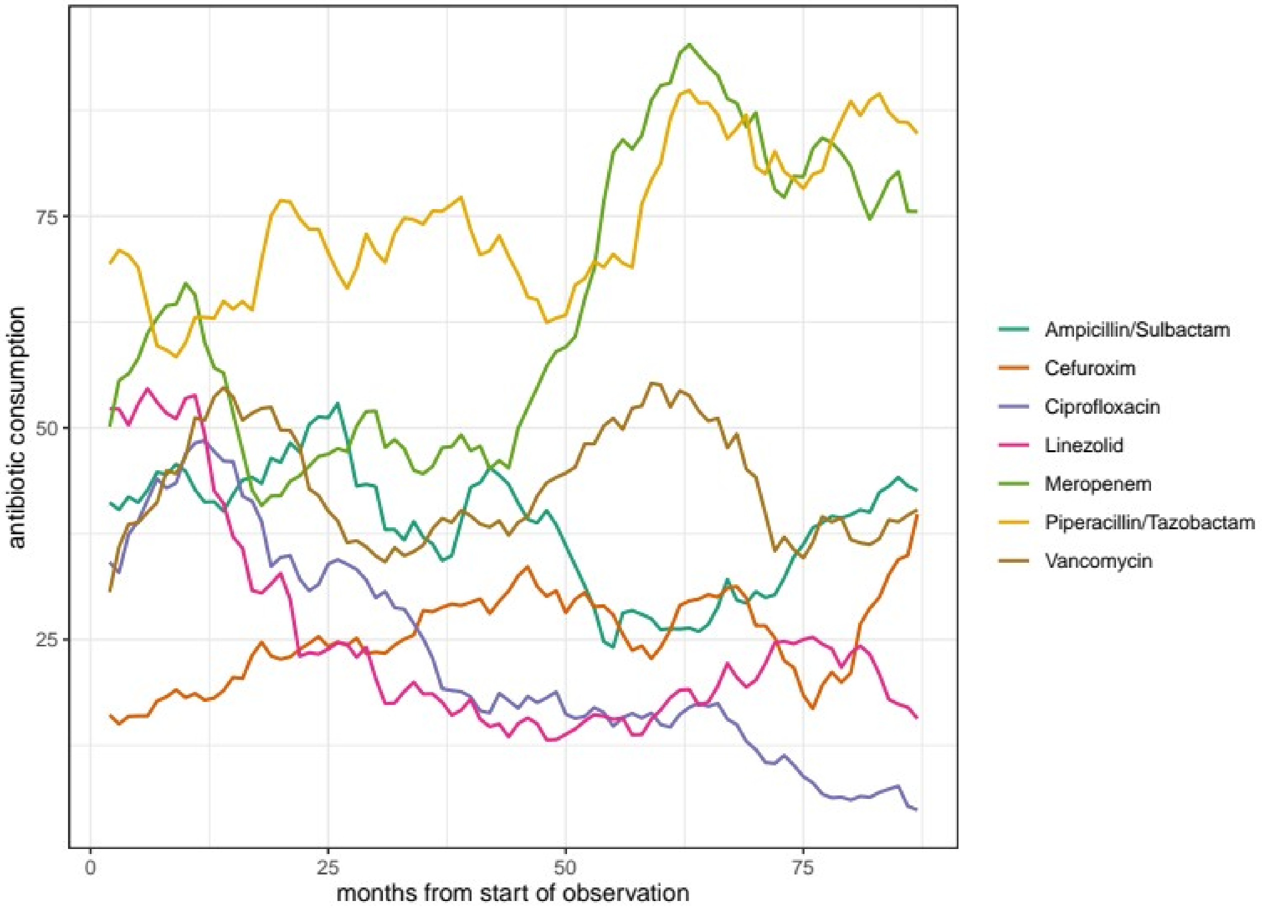

Figure S1: Monthly aggregated consumption of the 7 most frequently administered antibiotics. To ensure comparability, consumption figures are presented in a standardized format (see Methods section in the main text).

Supplement to Section 2.1.4 of the main document:
Antibiogram

In addition to the overall frequency of isolated pathogens for which antibiograms have been created, as discussed in Section 2.1.4, Figure S2 shows the time series of monthly aggregated detected cases for the eight most common pathogens. At around month 52, striking declines in the number of observed cases of Staphylococcus epidermidis, Enterococcus faecalis, and Staphylococcus aureus can be observed. The impact of the COVID-19 pandemic could be an explanation, albeit speculative.

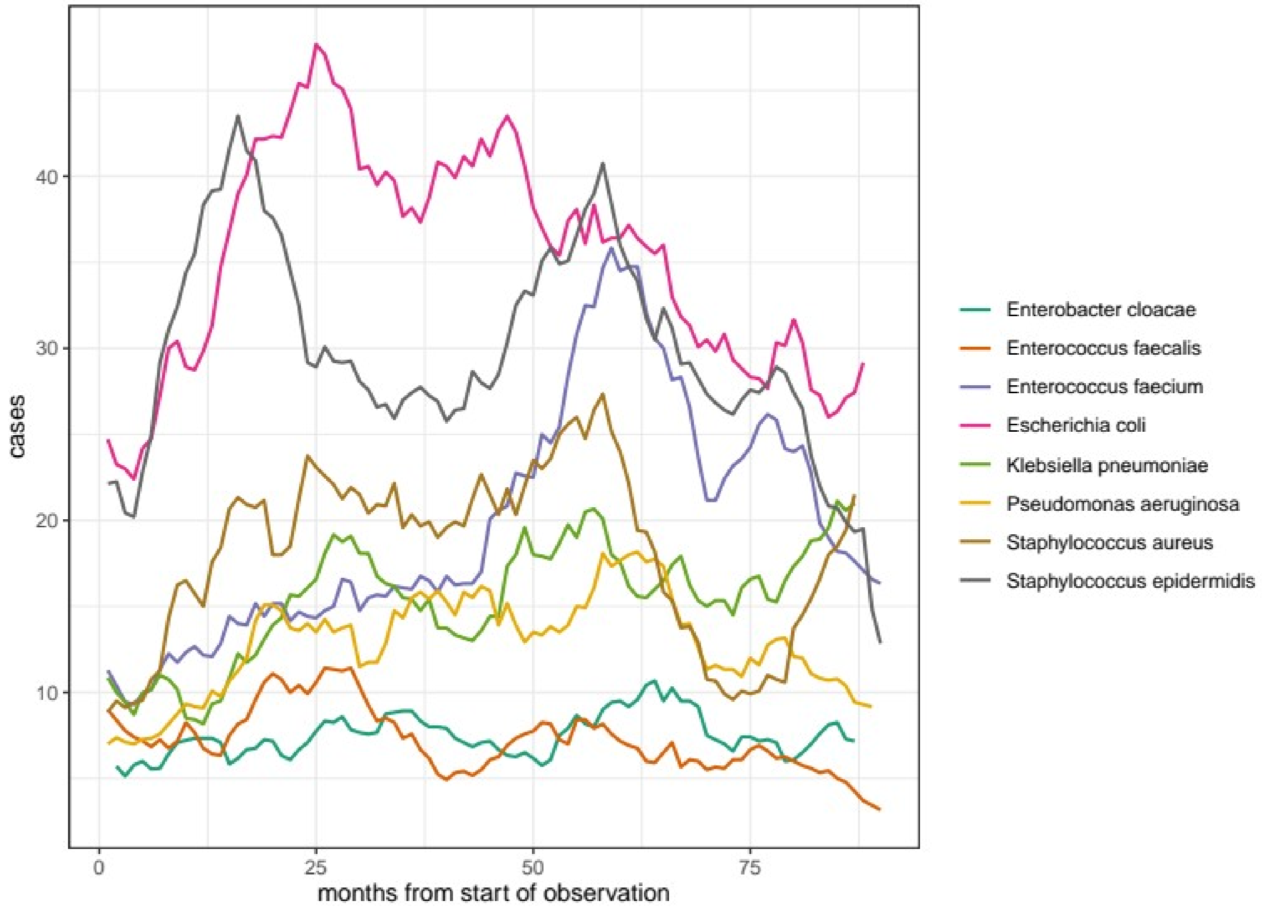

Figure S2: Time series of monthly aggregated detected cases for the eight most common pathogens.

Supplement to Section 2.4 of the main document:
Heterogeneity in antibiotic consumption

The different approaches to quantify heterogeneity, i.e., the different entropy measures, scale differently. In particular, Shannon entropy with its nonlinear scaling as a result of the logarithm used deviates considerably from AHI and Gini index especially in the case of very high and very low values. Strictly speaking, Shannon entropy is not defined when vanishing probabilities/proportions occur. On the other hand, the weightings of individual very large or very small proportions are particularly important in some applications in terms of their contribution to entropy. If the three heterogeneity measures are made comparable by means of a z-transformation, it becomes apparent that the three measures diverge particularly at very small values, depicted in Figure S3.

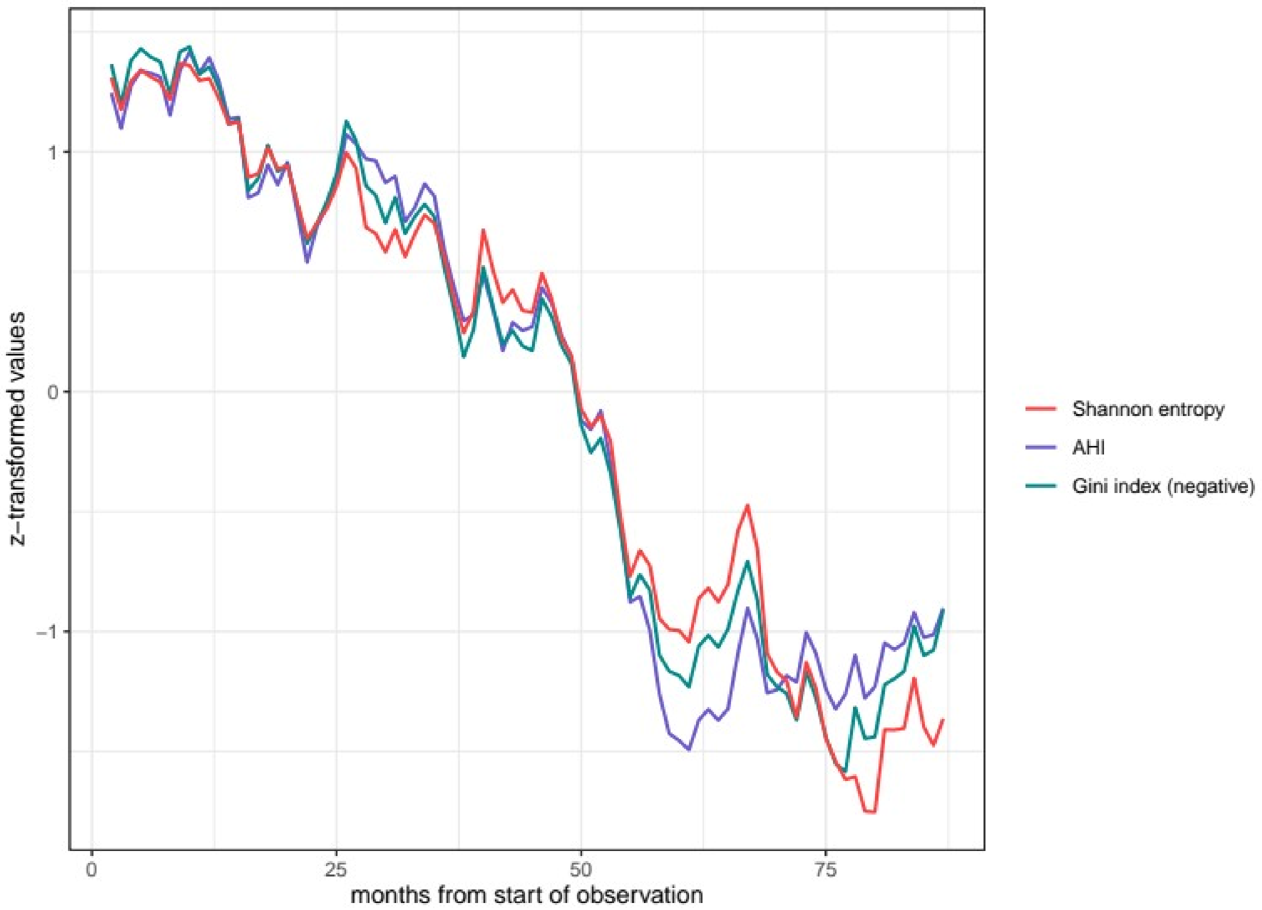

Figure S3: Z-transformed values of three heterogeneity time series as indicated.

Supplement to Section 2.5 of the main document:
Correlations between heterogeneity of antibiotic consumption and proportion of resistance

In the sense of a sensitivity analysis, the proportion of antibiograms detected as resistant for all combinations of the 8 most frequently investigated pathogens and the 7 most frequently administered antibiotics, p_R, is alternatively calculated by treating the intermediate category of antimicrobial sensitivity, I, as resistant. Note that I is defined as susceptible under increased dose of antibiotic administration. Here we treat I as if the challenged pathogen were resistant to the respective antibiotic. The time course of the newly calculated $p_R(t)$, analogous to that shown in Figure 8 in the main text, is depicted in Figure S4.

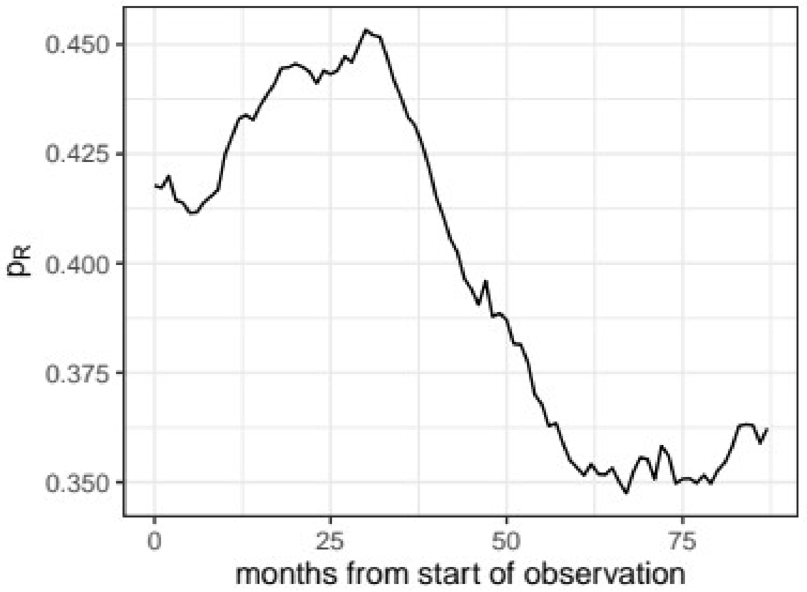

Figure S4: Proportion of antibiograms detected as resistant for all combinations of the 8 most frequently investigated pathogens and the 7 most frequently administered antibiotics with the intermediate category, I, treated as resistant, R.

As expected, the curve has shifted upward overall toward higher values. The shape of the trend is similar to the original curve. Somewhat surprising are the significantly less pronounced intermediate peaks.

Supplement to Section 2.5.2 of the main document:
Phase space construction reveals two-phase time course

The bi-phasic behavior, when being compared to the previous finding shown in Figure 10, is even more pronounced when I is set equal to R, i.e., an earlier and a later course in the trajectory in phase space appear very clearly separated as depicted in Figure S5.

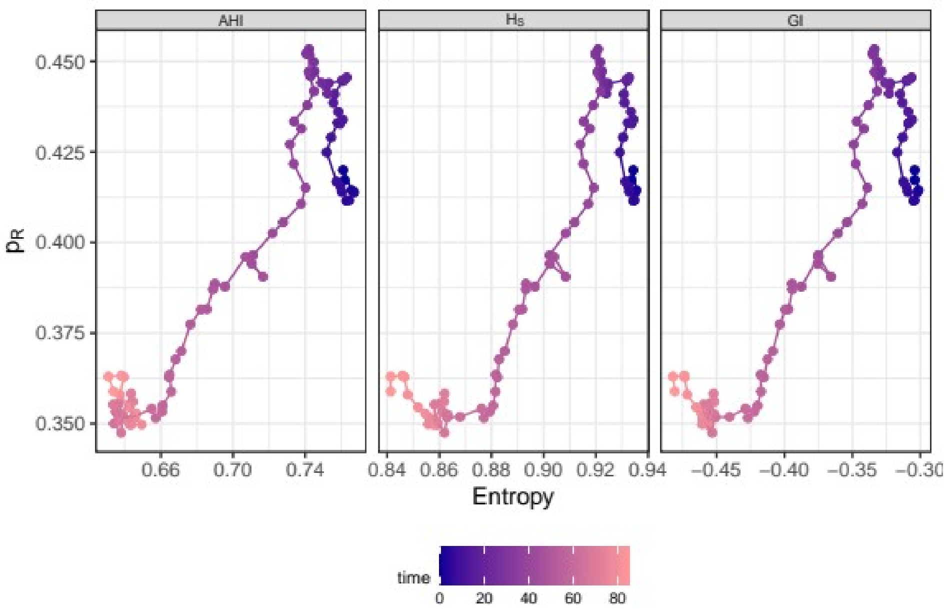

Figure S5: Trajectory in phase space spanned by AHI (left panel), Shannon entropy (middle panel), or negative Gini index (right panel), respectively and share of resistance drawn with color gradient by time starting at dark blue and ending at bright red. Share of resistance estimated unter the assumption that I corresponds to a resistant categegory.

The bi-phasic behavior can also be explored by means of a fragmented regression. To this end, a changepoint of the time variable is estimated such that the two regressions restricted to the interval before and the interval after this changepoint, respectively, maximizes the likelihood. The result of such a fragmented regression for both the antibiotic heterogeneity index, AHI, and the share of resistance, p_R, is shown in Figure S6 (upper panel: H_S; lower panel: p_R). For both curves, the intervals before the changepoint were approximated using second-order polynomials, and the intervals after the changepoint were approximated linearly.

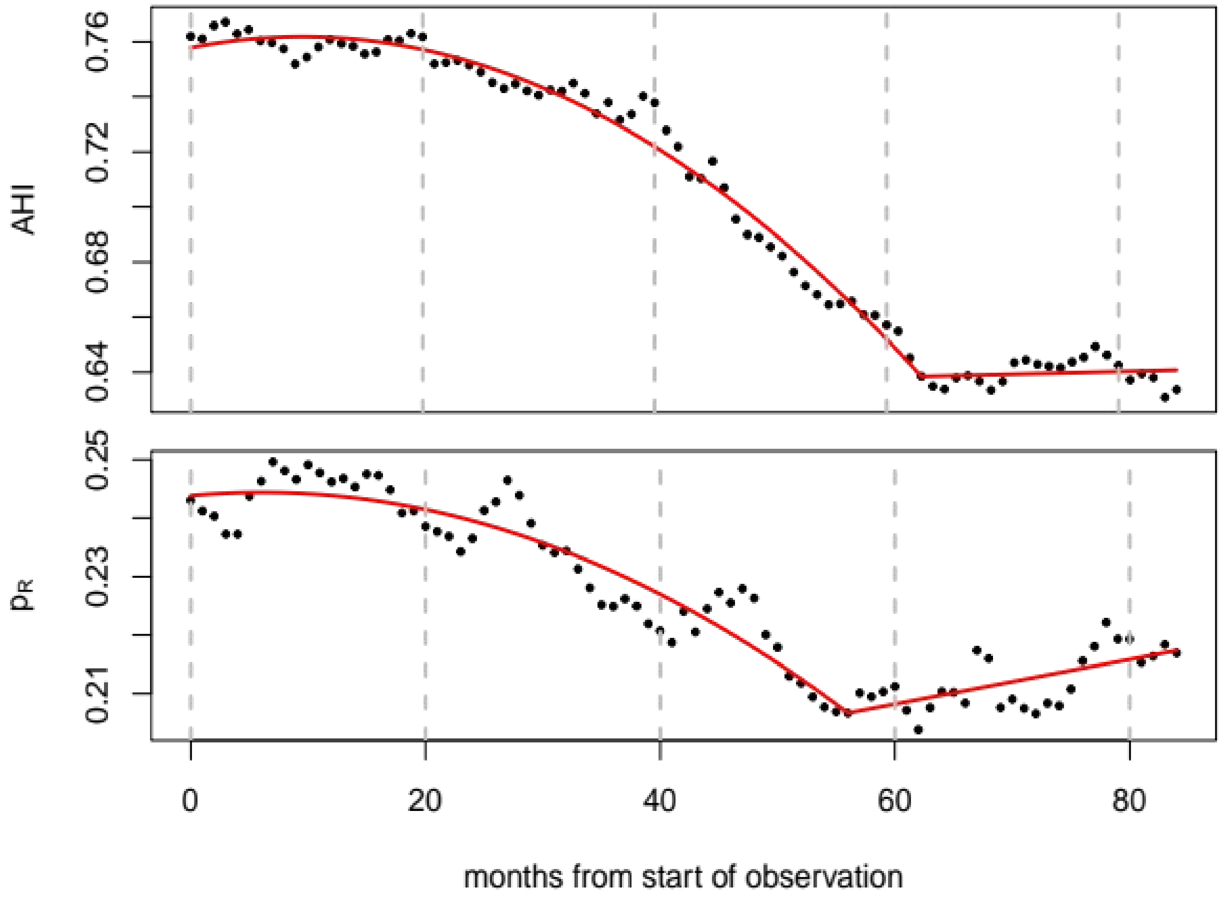

Figure S6: Fragmented regression of AHI and p_R, respectively, illustrates the bi-phasic behavior and similarity of shapes of both curves.
